# Antiproliferative Mechanisms of a Polyphenolic Combination of Kaempferol and Fisetin in Triple-Negative Breast Cancer Cells

**DOI:** 10.3390/ijms24076393

**Published:** 2023-03-29

**Authors:** Mohd. Afzal, Abdullah Alarifi, Abdalnaser Mahmoud Karami, Rashid Ayub, Naaser A. Y. Abduh, Waseem Sharaf Saeed, Mohd. Muddassir

**Affiliations:** 1 Department of Chemistry, College of Science, King Saud University, Riyadh 11451, Saudi Arabia; 2 Department of Science Technology Unit, King Saud University, Riyadh 11451, Saudi Arabia; 3 Restorative Dental Sciences Department, College of Dentistry, King Saud University, Riyadh 11545, Saudi Arabia

**Keywords:** breast cancer, flavonoids, kaempferol, fisetin, antioxidant

## Abstract

Herein, we investigate the combinatorial therapeutic effects of naturally occurring flavonoids kaempferol (K) and fisetin (F) on triple-negative breast cancer (TNBC: MDA-MB-231 cell line). Dose-dependent MTT assay results show that K and F exhibited cytotoxicity in MDA-MB-231 cells at 62 and 75 μM (IC_50_), respectively, after 24 h. However, combined K + F led to 40% and more than 50% TNBC cell death observed at 10 and 20 μM, respectively, which revealed the synergistic association of both. The combination of K and F was determined to be more effective in inhibiting cell viability than either of the agents alone. The morphological changes associated with significant apoptotic cell death were observed under a fluorescent microscope, strongly supporting the synergistic association between K and F. We also proposed that combining the effects of both polyphenols, as opposed to their individual effects, would increase their in vitro efficacy. Furthermore, we assessed the cell death pathway by the combinational treatment via reactive oxygen species-induced DNA damage and the mitochondrially mediated apoptotic pathway. This study reveals the prominent synergistic role of phytochemicals, which helps in elevating the therapeutic efficacy of dietary nutrients and that anticancer effects may be a result of nutrients that act in concert.

## 1. Introduction

Cancer—a rising threat (total global cancer 2020: 19.3 million)—is a multifactorial disease by which many people are shocked. Cancer—the big “C”—frequently tops the list as the most prevalent cause of death after cardiac arrest [1,2]. Amongst the various cancers that exist, breast cancer (BC) is the most commonly diagnosed in women, with a steadily increasing incidence, affecting human health and quality of life [3,4,5]. According to the World Health Organization (WHO), nearly 7.8 million women were diagnosed with BC in the year 2020 (WHO, 2021) [6]. Apart from epigenetic abnormalities, there are several other risk factors associated with BC, viz., hormonal imbalance, lack of breastfeeding, conceiving children at a later age, prolonged exposure to estrogen, and radiation [7,8,9]. Therapeutic regimes such as chemotherapy, radiotherapy, and immunotherapy have been employed to overcome tumor growth to a certain extent [10]. However, drawbacks such as adverse side effects, intrinsic and extrinsic resistance, and patient compliance limit their use [11,12]. Accordingly, new methods are required to counteract existing cancer chemotherapeutic drug profiles in terms of efficiency and toxicity. Phytochemicals (taxol analogs and vinca alkaloids such as vincristine, vinblastine, and podophyllotoxin) are known to serve as vibrant assets for cancer therapy [13,14]. These molecules act by regulating molecular pathways, which are associated with the growth of cancer.

Therefore, an important task for researchers today is in the field of medicinal chemistry is to examine combinatorial therapies (two or more drugs used to target multiple pathways) [15,16,17], which show promise in (i) lowering systemic dose-limiting toxicity and (ii) increasing survival rates of patients and (iii) exhibit superior chemical and pharmacological properties [18,19]. Combinatorial therapy exhibits many advantages over monotherapeutic drugs by providing a varied mode of action and influencing the long-term survival of the patient [20,21]. It is evident that single drugs require very high doses for potential activity, whereas lower doses of the same drug in combination with another drug may lead to improved chemopreventive effectiveness and translational impact [21,22]. Recently, Paul et al. studied the combination of two or more dietary compounds, which showed a synergistic effect in decreasing cancer cell viability in BC cell lines [20]. Aggarwal et al. showed that curcumin was capable of inhibiting BC metastasis of the lung by the expression of NF-κB-regulated genes [23]. Literature reports revealed that the synergistic effect of two flavonoids plays a key role in fighting against various cancers, including BC [24]. Therefore, combinatorial regimens undoubtedly intensify therapeutic goals over monotherapy because of their effectiveness in preventing and treating cancer.

In this context, natural products have regularly served as leads for the construction of various therapeutic agents with enhanced biological activity, particularly in combination with standard chemotherapeutics [25,26]. Flavonoids, a class of bioactive polyphenols, are not only known for their antioxidant properties but also possess many biological features, including anti-inflammatory [27], anticancer [28], anti-HIV [29,30], anticoagulant, antibacterial [31], and cardioprotective effects [32]. They are widespread in fruits, vegetables, and plant-derived sources such as green tea, wine, and cocoa-based products [26]. Numerous in vitro cytotoxicity studies have reported that flavonoid derivatives (either natural or synthetic) act as effects inhibitors of signaling pathways, which facilitate the growth, proliferation, and survival of cancer cells [26].

**Figure 1:**
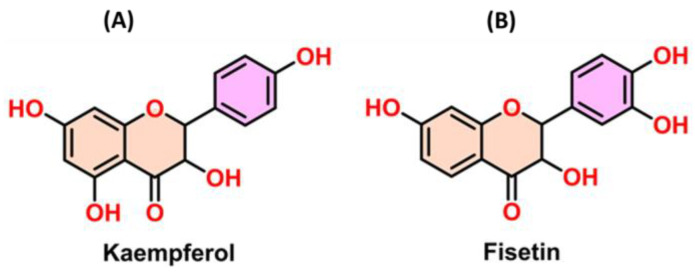
Molecular structure of (**A**) kaempferol and (**B**) fisetin.

Kaempferol (K) and fisetin (F) (Figure 1) are common dietary flavonols that exhibit certain therapeutic effects toward breast cancer [33]. Kaempferol arrests the G2/M phase of the cell cycle via the downregulation of CDK1 in BC cells [33]. Zhu et al. reported that the number of cells diminished considerably from 85.48% to 51.35% in the first-gap G1 phase and that the number of cells in the G2 phase was amplified significantly from 9.27% to 37.5% in TNBC after kaempferol application [34]. Similarly, Yi et al. reported that kaempferol could effectively induce the cleavage of poly(adenosine diphosphate-ribose) expression in BC cells by downregulating Bcl2 and promoting Bax protein expression [35].

Fisetin is another flavonoid of the same group involved in a series of cellular events, including cell apoptosis [36], which affects various signaling pathways [37]. Additionally, fisetin regulates the cell cycle and inhibits cyclin-dependent kinases (CDKs) in cancer cell lines [26]. Studies show that fisetin inhibits the proliferation of a series of breast cancer cells (4T1, MCF-7, and MDA-MB-231) in a dose- and time-dependent manner [38]. The cytotoxic potential of fisetin in MDA-MB-231 and MDA-MB-468 cells showed a significant decrease in the percentage of cells in the G1 phase of the cell cycle and a considerable increase in the percentage of cells in the G2/M stage.

In view of the aforementioned facts, herein, we attempted to investigate the combinatorial efficacy of kaempferol and fisetin in TNBC lines. We expected that combined treatment with these flavonols would be more effective than the additive effects of each drug alone.

## 2. Results

### 2.1. Dose Screening of Kaempferol and Fisetin for Combination Therapy

**Figure 2:**
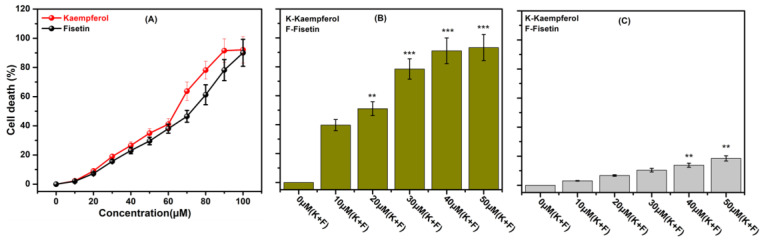
MTT assay showing (**A**) the effect of increasing doses (0–100 μM) of kaempferol (K) and fisetin (F) on the MDA-MB-231 cell line after 24 h, (**B**) the effect of K and F combination treatment on the MDA-MB-231 cell line after 24 h, and (**C**) the effect of K and F combination treatment on the MCF-10A cell line after 24 h. The significant results with *p* values < 0.05 are labelled as follows (**B**): ** control versus 20 μM (K) + 20 μM (F); *** control versus 30 (K + F), 40 (K + F), and 50 μM (K + F). Statistical significance: ** *p* < 0.01, and *** *p* < 0.001.

Using an MTT assay, we first screened the cytotoxicity of K and F individually (Figure 2A). In TNBC cells (MDA-MB-231), K and F treatments were applied at different concentrations (0 to 100 μM). It was found that both phytochemicals showed cytotoxicity at concentrations beyond 20 μM. In the individual case of K after 10 and 20 μM treatments, the percentage of death cell was 2.24 ± 0.17 and 9.04 ± 0.93, respectively. On the other hand, in the individual case of F, after 10 and 20 μM treatments, the percentage of death cell was 1.93 ± 0.1 and 7.28 ± 0.62, respectively. Then we applied a 10 μM concentration of K and a 10 μM concentration of F in combination to observe the synergistic efficacy, which is denoted as 10 μM (K + F). Concentrations of 20, 30, 40, and 50 μM (K + F) were also included in the MTT assay on the MDA-MB-231 cell line to determine the IC_50_ concentration in the case of combination therapy (Figure 2B). Colorimetric data indicate that 39.71 ± 3.83% and 51.03 ± 4.78% of cells died after 24 h of 10 and 20 μM (K + F) treatment, respectively. In the case of treatment with only K and only F, the IC_50_ was 64.23 ± 6.7 and 73.26 ± 7.21, respectively. Subsequently, at concentrations of 30 μM (K + F), 40 μM (K + F), and 50 μM (K + F) cell death was increased after 24 h of incubation. A cytotoxicity study of the K and F combination treatment was also conducted in the MCF-10A cell line at different doses: 10 μM (K + F), 20 μM (K + F), 30 μM (K + F), 40 μM (K + F), and 50 μM (K + F) (Figure 2C). The cytotoxicity data showed that there was no significant cytotoxicity up to a 30 μM (K + F) dose of the combination treatment in the MCF-10A cell line. We investigated the nontoxic concentration of K and F individually to understand the synergistic efficacy. Therefore, all further studies were carried out with two different sets of concentrations: 10 μM (K + F) and 20 μM (K + F).

### 2.2. Quantification of Apoptosis and Necrosis

**Figure 3:**
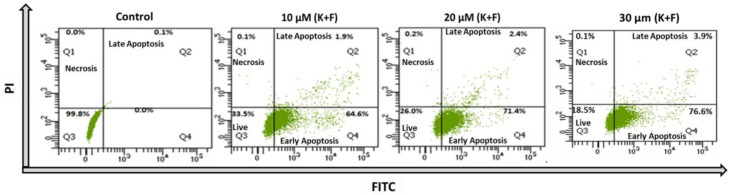
Annexin V-FITC/PI-positive MDA-MB-231 cells 24 h after treatment with 10, 20, and 30 μM (K + F) according to flow cytometry showing different stages of the cells.

The apoptotic study was evaluated using an annexin V-FITC and propidium iodide (PI) kit with the above dose combination of K and F for treatment of MDA-MB-231 cells. The flow cytometric analysis established that the percentages of live, early apoptotic, late apoptotic, and necrotic cells were 99.8%, 0%, 0.1%, and 0%, respectively, relative to untreated cells. The activity of K (50 μM) alone showed apoptosis in the MDA-MB-231 cell line [34], whereas treatment with F alone at two different concentrations (25 and 50 μM) showed a similar effect on the MDA-MB-453 breast cancer cell line [39]. However, in both the cases, a higher concentration was required for potential activity. Based on the preliminary MTT assay, doses of 10 and 20 μM (K + F) were selected for combination treatment for further cell biological experiments. In the case of MDA-MB-231 cells treated with 10 μM (K + F), the percentages of live, early apoptotic, late apoptotic, and necrotic cells were 33.5%, 64.6%, 1.9%, and 0.1% respectively, after 24 h of treatment, whereas in the case of cells treated with 20 μM (K + F), the percentages of live, early apoptotic, late apoptotic, and necrotic cells were 26%, 71.4%, 2.4%, and 0.2%, respectively. Consequently, the percentages of live, early apoptotic, late apoptotic, and necrotic cells were also calculated after treatment with 30 μM (K + F). The flowcytometric data also reflected that the early and late apoptotic percentages were increased compare to 10 and 20 μM (K + F) treatments, confirming that cell death is due to apoptosis (Figure 3).

### 2.3. Mitochondrial Membrane Potential Change (ΔΨ_m_)

**Figure 4:**
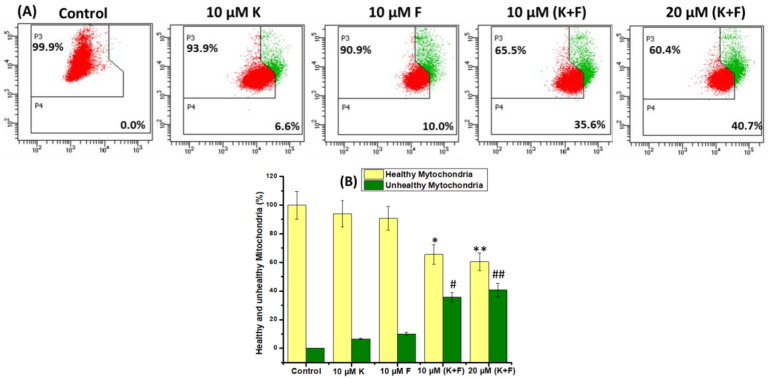
(**A**) Mitochondrial membrane potential measurement of MDA-MB-231 cells 24 h after treatment with 10 μM K, 10 μM F, 10 μM (K + F), and 20 μM (K + F) by flow cytometry. (**B**) Bar plot of (**A**) healthy mitochondria; * denotes control versus 10 μM (K + F); ** denotes control versus 20 μM (K + F) unhealthy mitochondria; # denotes control versus 10 μM (K + F); ## denotes control versus 20 μM (K + F). Statistical significance: * *p* < 0.05, ** *p* < 0.01.

Disruption of mitochondria is a sign of apoptotic cell death [40]. Changes in the mitochondrial membrane potential (ΔΨ_m_) serve as an indicator of mitochondrial function because of the opening of the mitochondrial permeability transition pores (MPTPs) and are associated with early apoptotic fate [41]. Figure 4A displays the membrane potential analysis of the MDA-MB-231 cells treated with two different concentrations of K and F. The flow cytometric outcome shows that in the case of the untreated control, 0% of the cell population showed changes in ΔΨm, whereas in the case of cells treated with 10 μM K, 10 μM F, 10 μM (K + F), and 20 μM (K + F), the ΔΨm-changed population increased to 6.6, 10, 35.6, and 40.7%, respectively. A bar plot was generated added for improved understanding (Figure 4B).

### 2.4. Quantification of Intercellular Reactive Oxygen Species

**Figure 5:**
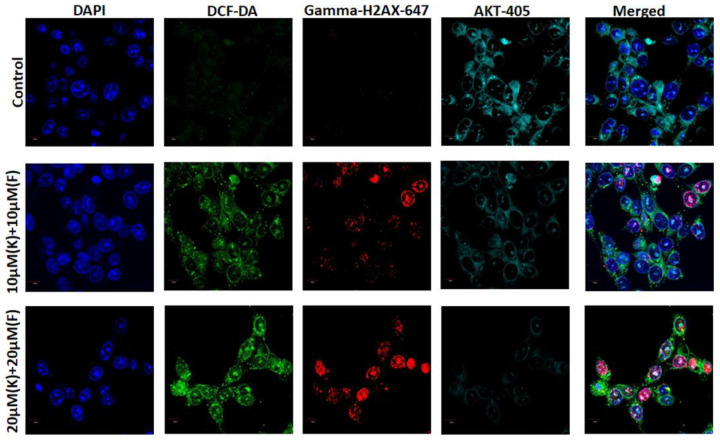
ROS, γ-H2AX, and Akt expressed after treatment with 10 and 20 μM (K + F) against MDA-MB-231 cells after 24 h by confocal microscopy; DAPI was used as a nuclear stain.

Oxidative stress in cancer cells is produced by K and F as a result of an imbalance between the production and accumulation of individual reactive oxygen species (ROS) [42]. Intracellular ROS were identified using the cell-permeant dye 2′,7′-dichlorofluorescein diacetate (DCF) [43]. The amount of ROS produced within the cells was directly correlated with the relative DCF fluorescence maximum at 535 nm upon excitation at 485 m. After treatment with 10 and 20 μM (K + F), the microscopic images confirmed that with an increase in K and F concentrations, the green DCF fluorescence increased (Figure 5).

### 2.5. K and F Induced Phosphorylation of H2AX in MDA-MB-231 Cells

It is well known that apoptosis is a highly synchronized procedure that is essential for the survival of multicellular organisms [44]. It is evident that highly reactive, oxygen-containing molecules generated under excess cellular levels of ROS can induce DNA damage and ultimately lead to cell death (apoptosis/necrosis) [45]. The activation of phosphorylation of the histone variant H2AX during DNA damage response (DDR) generates γH2AX, which is required for the assembly of DNA repair proteins [46]. The formation of γ-H2AX is a characteristic step for DNA damage, which induces the activation of downstream signals associated with apoptosis. In the confocal images, it was observed that the red foci were increased with an increasing concentration of K and F combination treatment (Figure 5).

### 2.6. Crosstalk between Akt, γ-H2AX, and Reactive Oxygen Species (ROS) after Treatment with K and F

Fisetin has been shown to play an important role in regulating the PI3K/Akt pathway [47]. It has been reported that F and K downregulated Akt in astrocytes in a concentration-dependent manner but had no effect on p38 expression and phosphorylation. Therefore, the expression of Akt was evaluated with DCF-DA and γ-H2AX. The results confirm that the expression of Akt was decreased, whereas the expression of DCF-DA and γ-H2AX was found to be increased with increasing concentrations of K and F combination treatment (Figure 5).

### 2.7. K and F Activated Bax and Cytochrome c Expression

**Figure 6:**
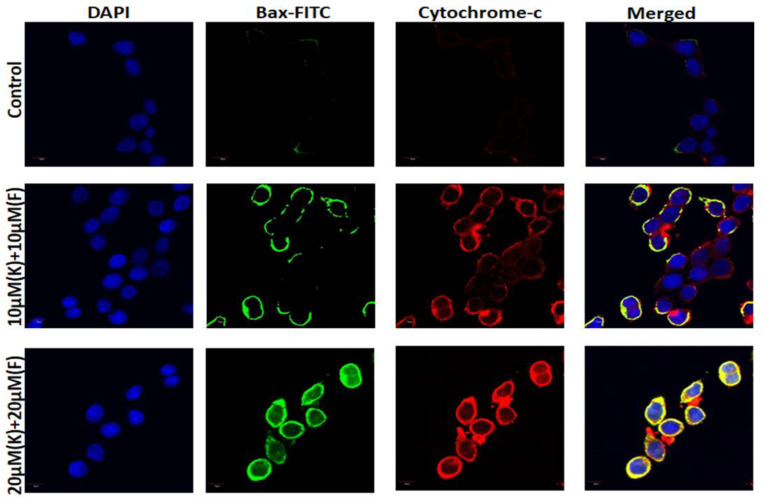
Expression of Bax and cytochrome c after treatment with 10 and 20 μM (K + F) against MDA-MB-231 cells after 24 h by confocal microscopy; DAPI was used as nuclear stain.

In order to confirm the Akt-induced activation of apoptosis in the K and F combination treated condition, immunofluorescence was carried out [48]. The expression of Bax and cytochrome c was evaluated by confocal microscopy with 10 and 20 μM (K + F) doses. The images further confirmed that the expression of Bax and cytochrome *c* was increased in a dose-dependent manner after 24 h of treatment (Figure 6), confirming that apoptosis is linked with mitochondria-dependent pathways.

### 2.8. The Effect of Combination of K and F Treatment on Caspase 3/9 Activation in MDA-MB-231 Cells

**Figure 7:**
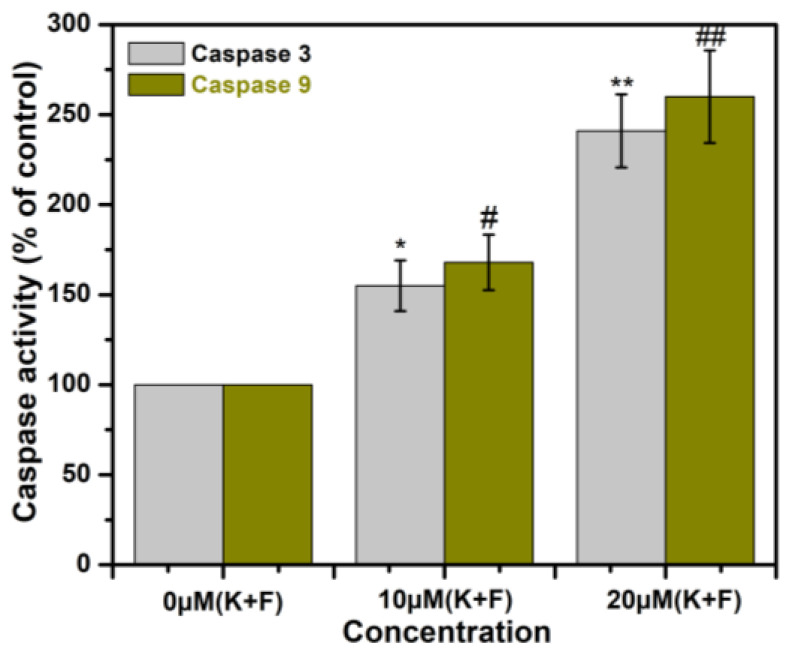
The level of active caspase-3 and caspase-9 after 10 and 20 μM (K + F) treatments for 24 h. * and # has been used to compare the caspase-3 and caspase-9 activity with control. Statistical significance: */# *p* < 0.05, **/## *p* < 0.01.

Caspases are a class of endoproteases that cause apoptosis by cleaving particular enzymes [49]. Caspase activation, which is responsible for triggering the start of apoptosis, can be brought on by the upregulation of Bax/cytochrome c. In accordance with the data, caspase 3/9 activity was gradually increased in MDA-MB-231 cells after treatment with the combination of K and F in a dose-dependent manner after 24 h of incubation (Figure 7). This experiment was performed three times.

## 3. Materials and Methods

### 3.1. Cell Lines and Chemicals

Human TNBC (MDA-MB-231) and human breast epithelial (MCF-10A) cell lines were obtained from ATCC, USA. Cell culture constituents such as fetal bovine serum (FBS), DMEM, penicillin–streptomycin−neomycin (PSN) antibiotic, EDTA, and trypsin were purchased from Gibco (Grand Island, New York, NY, USA). Kaempferol (CAS number 520-18-3) and Fisetin (CAS number 528-48-3) were purchased from Sigma-Aldrich (St. Louis, MO, USA). Antibodies were obtained from Santa Cruz Biotechnology (Dallas, TX, USA) and eBioscience (San Diego, CA, USA).

### 3.2. Cell Culture and Cytotoxicity Assay

Cells were separately grown in a humidified atmosphere (5% CO_2_) using DMEM consisting of 10% FBS and 1% PSN antibiotics. Cells were cultured in phosphate-buffered saline (PBS) using EDTA (0.5 mM) and trypsin (0.25%) after reaching 75–80% confluence and planted at the preferred density, then allowed to re-establish their equilibrium for one day following a previously reported procedure [50].

### 3.3. Cell Viability

Culture of both cells was carried out by a trypsinization procedure to a final concentration of 3 × 10^4^ cells/mL. Cells were seeded at a required density in a 96-well plate. Initially, K and F were treated separately at different concentrations with cancer cell lines [51]. Then, 10 μL of MTT solution (4 mg/mL) was added to each well, and the plate was incubated for 4 h. The reduced dark blue formazan crystals were dissolved in DMSO. The same protocol was used for the following equal dose combinations of K and F using appropriate controls; 10, 20, 30, 40, and 50 μM. The combination concentrations were also treated in MCF-10A to determine whether there was any cytotoxicity toward normal breast cell lines. The absorbance was monitored at 595 nm using a microplate reader (Emax, Molecular Device, Brea, CA, USA). The experiments were repeated in triplicate, and the average value is reported. In our case, the combined effect of K and F was examined against a TNBC cell line (MDA-MB-231), and MCF-10A was used as healthy breast cell line model.

### 3.4. Quantification of Apoptosis

Ab annexin V-FITC apoptosis detection kit (Calbiochem, San Diego, CA, USA) was used to analyze apoptosis [52]. During this procedure, the cells treated with K and F were washed and stained with annexin V-FITC in accordance with the manufacturer’s instructions. The concentration of K and F was 10 μM each for the combination treatment, whereas 20 μM (K + F) was used as the treatment concentration of K and F for individual treatments. K and F were combined with DMSO to make a 1 mM stock solution of K + F. Then, the treatment was administered to reach 10 μM (K + F) and 20 μM (K + F). The untreated cells were taken as a control. The percentages of live, apoptotic, and necrotic were estimated by flow cytometry (BD LSRFortessa, San Jose, CA, USA).

### 3.5. Measurement of Mitochondrial Membrane Potential

The cells treated with the combination of K and F were subjected to incubation with the cationic dye JC-1 (5,5′,6,6′-tetrachloro-1,1′,3,3′ tetraethyl benzimidazolyl carbocyanine iodide) and analyzed using a flow cytometer (BD LSRFortessa^TM^ San Jose, CA, USA). Cells left untreated served as the control group. JC-1 exhibited potential-dependent aggregation in mitochondria, leading to a green to red fluorescence emission (590 nm) [53]. Based on the subsequent fluorescence of JC-1, the variation in fluorescence intensity showed the fraction of depolarized and hyperpolarized mitochondria. The experiments were repeated in triplicate, and average values are reported.

### 3.6. Reactive Oxygen Species (ROS) Measurement

MDA-MB-231 cultured cells (2 × 10^5^) in a 6-well culture plate were treated with a combination of K and F for 24 h. Cells left untreated served as the control group [54]. Then, the cells were washed twice with PBS and diluted with DCFH-DA (1:1000). The samples were incubated in the dark for 20 min at 37 °C, then subjected two 10 min rinses in PBS (0.01 M). The coverslips containing cells were mounted with the Prolong antifade reagent after being counterstained for 10 min with 6-diamidino-2-phenylindole (DAPI) (Molecular Probe, Eugene, OR, USA). The experiments were repeated in triplicate.

### 3.7. Confocal Microscopy

The MDA-MB-231 cells treated with combined doses of K and F were incubated for 24 h, followed by 10 min washes in 0.01 M PBS twice. The coverslips containing MDA-MB-231 cells were incubated for 1 h in a blocking solution made up of 2% normal bovine serum and 0.3% Triton X-100 in PBS. The appropriate primary antibody was used to incubate the slides overnight at 4 °C following blocking (Gamma H2AX, AKT, Bax and cytochrome c) [55]. The primary tagged antibody for Gamma H2AX was Alexa Fluor 647, and the secondary tagged antibodies for Akt, Bax, and cytochrome c were 405, FITC, and Alexa Fluor 647, respectively. In the blocking solution, secondary antibodies were diluted 1:100 before being incubated for 2 h. The slides were fixed using Prolong antifade reagent after being counterstained for 10 min with DAPI (Molecular Probe, Eugene, OR, USA).

### 3.8. Measurement of Caspase 3 and 9 Activity

The activities of caspase 3 and 9 enzymes were determined in accordance with the manufacturer’s instructions using a colorimetric assay kit (Bio Vision Research Products, Mountain View, CA, USA), [56]. Using an ELISA reader, caspase activity was measured at 405 nm. The experiments were repeated in triplicate.

### 3.9. Statistical Analysis

Data are reported as mean ± standard error of mean (SEM). Statistical significance and differences among the groups were calculated by the use of one-way analysis of variance (ANOVA) using OriginPro 8.0 software (San Diego, CA, USA). *p* values < 0.05 were considered significant. Post hoc examinations were carried out to determine the presence of statistical significance.

## 4. Discussion

The objective of this study was to examine the combined efficacy of two major flavonoids (K and F) in a TNBC cell line (MDA-MB-231). According to earlier research, both flavonoids have anti-inflammatory and anticancer characteristics [57]. The efficacy of combination treatment was the focus of the current study. In MTT experiments, cotreatment of MDA-MB-231 with K and F consistently resulted in increased suppression of cell proliferation. The findings of this investigation strongly suggest that the combination of K and F therapy had a synergistic impact in reducing cancer cell proliferation. Due to their low absorption, K and F are probably more effective when used topically. The synergistic effect of K and F was evaluated using MDA-MB-231 cells. An MTT assay was also conducted in the MCF-10A cell line with doses of 10, 20, 30, 40, and 50 μM (K + F). These data further confirmed that up to concentrations of 40 μM (K + F), no significant cytotoxicity was observed. Therefore, for subsequent studies, doses of 10 and 20 μM (K + F) were selected, and treatment was administered for 24 h. K and F have previously been reported to exhibit anticancer potential against MDA-MB-231 cell lines at 20 μM and 40 μM concentrations, respectively [25,58]. Here, the synergistic effect of both K and F was evaluated against the MDA-MB-231 cell line. Based on the results of the MTT assay, 10 μM (K + F) and 20 μM (K + F) doses were selected for further cell biological assays.

A comprehensive flow cytometric analysis was implemented to assess the mechanism of cell death governed by the combined treatment strategy employing K and F. During the course of cell death, a phospholipid constituent is externalized, which is present across the cell membrane [59]. Annexin V is a phosphatidylserine (PS)-specific binding protein that can be used to detect apoptotic cells when conjugated with a specific fluorophore. The extent of apoptosis and necrosis was observed using a DNA-binding dye (PI), along with annexin V. The flow cytometric method was also employed using annexin V-FITC/PI to explore the mechanism of the effect of K and F doses on MDA-MB-231 cell lines. The percentage of viable cells after the combine effect of K and F was found to be 33.5% and 26% for doses of 10 μM (K + F) and 20 μM (K + F), respectively. However, the percentage of apoptotic (early and late) cells gradually increased in a concentration-dependent manner following combined doses of K and F. Throughout the apoptosis process, a decrease in mitochondrial membrane potential (MMP) was observed [60]. Therefore, after confirmation of apoptosis, we checked the MMP by flow cytometric analysis and found that the FITC+ cell population was increased with increasing concentration of the combination treatment. Then, ROS generation was evaluated by confocal microscopy by DCF-DA, which also showed green emission with an increasing concentration. These microscopic images confirmed that oxidative stress increased in response to the synergistic effect of K and F.

Several reports have established that oxidative stress can induce H2AX phosphorylation [61]. At serine139, phosphorylated gamma histone H2AX (γH2AX) was generated, which is sensitive to radiation-induced DNA damage is a probable marker for the recognition of direct DNA damage with other therapeutic agents [62]. The expression of γH2AX was checked by confocal microscopy; expression was increased after K and F treatment.

There are several protein-encoded signaling pathways (PTEN, PI3K, PKB, and Akt) in breast cancer that are involved in the DNA repair mechanism and maintaining the genomic integrity for cell growth and survival [63]. The activation of primordial follicles via this pathway may result in a DNA damage response (DDR). Therefore, we also checked the expression of Akt after treatment with the combination of K and F, which was found to be decreased as the concentration was increased after 24 h of treatment.

We also observed the expression of mitochondrial apoptotic hallmark proteins [64] Bax and cytochrome *c* by confocal microscopy. The microscopic images established that the expression of both proteins was increased. There was a close correlation between the duration of cytochrome *c* release from the mitochondria and caspase activation. Thus, we estimated the expression of caspase-3 and -9 using ELISA, and high expression was observed after treatment with a combination of K and F. In summary, a combination of K and F has considerable anticancer potential via oxidative stress-mediated DNA damage, in which mitochondria play an important role.

## 5. Conclusions

In conclusion, we determined the synergistic anticancer effect of two flavonoids (K and F) on a TNBC cancer cell line (MDA-MB-231) through nuclear alterations. Combinatorial studies and fluorescence microscopic examination revealed a strong synergistic role of these flavonoids in MDA-MB-231 at two different concentrations. The results show that combined treatment with these two flavonols is more potent in reducing cell proliferation than either drug alone, resulting in the selective enhancement of the cytotoxic effect in breast cancer cells. These effects may have arisen via attenuation of the activation of p-Akt and suppression of the PI3K/Akt pathway because of ROS-induced DNA damage and the mitochondria-mediated apoptotic pathway. Therefore, our results warrant further preclinical confirmation of in vivo significance to determine clinical utility.

## Data Availability

Not applicable.
